# Profile observations of the Arctic atmospheric boundary layer with the BELUGA tethered balloon during MOSAiC

**DOI:** 10.1038/s41597-023-02423-5

**Published:** 2023-08-10

**Authors:** Christian Pilz, Michael Lonardi, Ulrike Egerer, Holger Siebert, André Ehrlich, Andrew J. Heymsfield, Carl G. Schmitt, Matthew D. Shupe, Birgit Wehner, Manfred Wendisch

**Affiliations:** 1 Atmospheric Microphysics Department, Leibniz Institute for Tropospheric Research (TROPOS), Leipzig, Germany; 2 Leipzig Institute for Meteorology (LIM), Leipzig University, Leipzig, Germany; 3 National Center for Atmospheric Research (NCAR), Boulder, Colorado USA; 4 Geophysical Institute, University of Alaska, Fairbanks, Alaska USA; 5 Cooperative Institute for Research in Environmental Sciences (CIRES), University of Colorado, Boulder, Colorado USA; 6 Physical Sciences Laboratory (PSL), National Oceanic and Atmospheric Administration (NOAA), Boulder, Colorado USA; 7 Present Address: National Renewable Energy Laboratory (NREL), Golden, Colorado USA

**Keywords:** Atmospheric dynamics, Atmospheric dynamics

## Abstract

During the Multidisciplinary drifting Observatory for the Study of Arctic Climate (MOSAiC) expedition, the Balloon-bornE moduLar Utility for profilinG the lower Atmosphere (BELUGA) was deployed from an ice floe drifting in the *Fram Strait* from 29 June to 27 July 2020. The BELUGA observations aimed to characterize the cloudy Arctic atmospheric boundary layer above the sea ice using a modular setup of five instrument packages. The *in situ* measurements included atmospheric thermodynamic and dynamic state parameters (air temperature, humidity, pressure, and three-dimensional wind), broadband solar and terrestrial irradiance, aerosol particle microphysical properties, and cloud particle images. In total, 66 profile observations were collected during 33 balloon flights from the surface to maximum altitudes of 0.3 to 1.5 km. The profiles feature a high vertical resolution of 0.01 m to 1 m, including measurements below, inside, and above frequently occurring low-level clouds. This publication describes the balloon operations, instruments, and the obtained data set. We invite the scientific community for joint analysis and model application of the freely available data on PANGAEA.

## Background & Summary

The Arctic environment has undergone substantial changes over the last decades, including a dramatic loss in sea ice^1^. Between 1979 and 2021, the Arctic mean air temperature increased three to four times faster than the global mean temperature, depending on the considered southern boundary of the Arctic^2^. This accelerated Arctic warming is known as Arctic amplification and it results from local and remote feedback mechanisms in response to global warming^3^. While the surface albedo and lapse rate feedback are thought to be the main drivers^4^, further complex atmospheric boundary layer (ABL) processes are considered to contribute significantly to Arctic warming^5^.

The summertime Arctic ABL above sea ice is often neutrally stratified and dominated by low-level liquid or mixed-phase clouds^6^. Capping temperature inversions near the cloud top represent a barrier for the vertical transport of heat, moisture, aerosol, and trace gases between the free troposphere and the ABL. Radiative cooling at the cloud top induces entrainment from aloft and turbulent mixing inside the ABL. However, the cloud-mixed layer is often decoupled from the surface when turbulence is vertically discontinuous^7^. The magnitude of terrestrial cooling in Arctic mixed-phase clouds depends on cloud microphysical properties^8^ that, in turn, are affected by the limited availability of aerosol particles acting as cloud condensation nuclei (CCN) or ice nucleating particles (INP)^9,10^. The long-range transport of absorbing particles may play an essential role by causing atmospheric heating or decreasing surface albedo when deposited on snow and ice^11^.

**Fig. 1:**
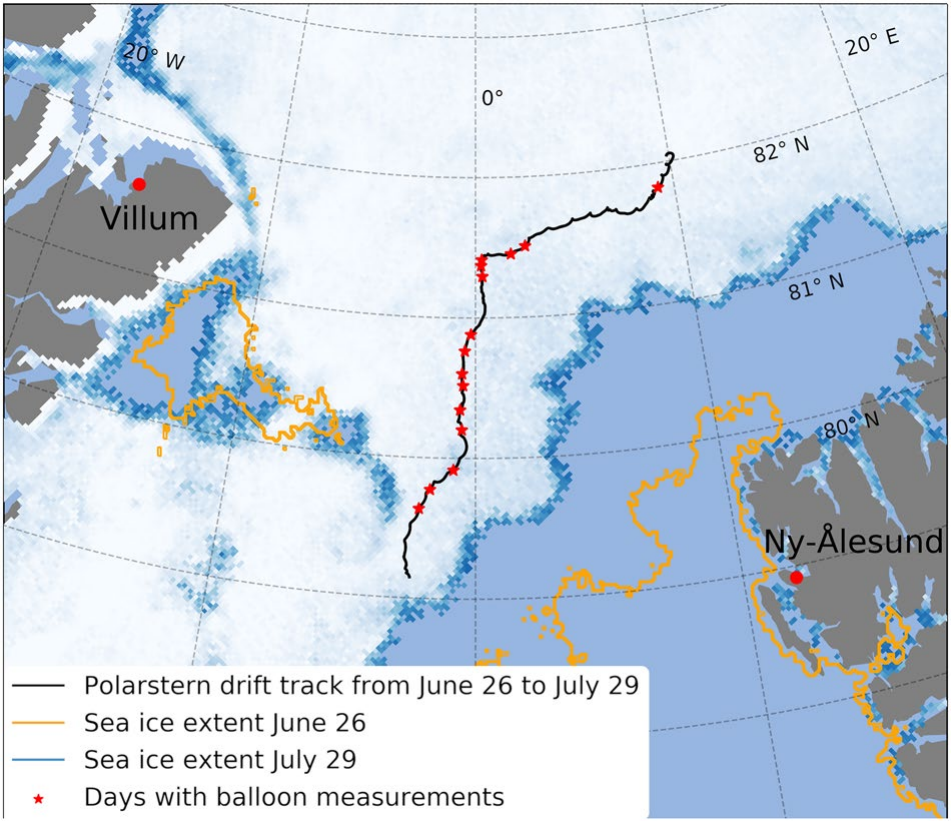
Drift pattern of RV *Polarstern* with the ice floe during the BELUGA deployments (black line) with each day of balloon observation highlighted as red stars. The sea ice extent was derived from the MODIS-AMSR2 data set^42^.

Despite significant efforts and improvements over the past two decades, models still have significant uncertainties that inhibit our ability to understand the impact of ABL processes on Arctic amplification^4^. The essential measurements needed to overcome these issues, particularly above the sea ice, are still sparse. Therefore, a vast international effort was undertaken with the Multidisciplinary drifting Observatory for the Study of Arctic Climate (MOSAiC) from September 2019 to October 2020. The Research Vessel (RV) *Polarstern*^12^ supported by the research expedition into the central Arctic Ocean^13^. During the latter part of MOSAiC, the Balloon-bornE moduLar Utility for profilinG the lower Atmosphere^14^ (BELUGA) system was deployed from the research camp on the sea ice floe, from 29 June to 27 July 2020. BELUGA was operated on 33 flights resulting in 66 high-resolution profiles up to an average of 1 km height on 14 days. During that time, RV *Polarstern* drifted with the ice floe from the Arctic Ocean at 82.2°N, 10.1°E into the *Fram Strait* at 79.1°N, 2.4°W (Fig. 1).

A modular setup of five custom-built instrument packages was deployed with BELUGA: an extended meteorological package (EP), an ultrasonic anemometer package (UP), a broadband radiation package (BP), a Cubic Aerosol Measurement Platform^15^ (CAMP), and a Video Ice Particle Sampler^16^(VIPS). A first interpretation of the BELUGA observations setting them in context with continuous ground-based atmospheric remote sensing observations made onboard RV *Polarstern* was shown by Lonardi *et al*.^17^. Additional *in situ* measurements of atmospheric trace gases^18^ and airborne observations by uncrewed aerial vehicles (UAV)^19^, a smaller tethered balloon, and the helicopter-borne platform HELiPOD are available for combined data analysis. Table 1 gives an overview of the instrument packages and further specifications. Where it applies, we will refer to the detailed instrument description in Egerer *et al*.^14^ and Pilz *et al*.^15^.

**Table 1:** Overview of the operated instrument packages and the measured main quantities that are part of the data set.

Instrument package	Instrument	Model	Manufacturer	Measured quantity	Measurement range	Measurement uncertainty	Temporal resolution
Extended meteorological package (EP)	Radiosonde	DFM-17	GRAW	Temperature (*T*) Pressure (*p*) Relative humidity (RH)	−90 to +60 °C 1 to 1100 hPa 0 to 100%	0.2 °C 1 hPa <3%	1 Hz
	Pitot-static tube	custom		horizontal wind speed (*u*)	1.8 to 20 ms^−1^	0.2 ms^−1^	1 Hz
	3-Axis compass	HMC6343	Honeywell	Wind direction (D)	0 to 360°	2°	1 Hz
Ultrasonic anemometer package (UP)	Ultrasonic anemometer	uSonic-3 Class A	Metek	3-d wind (*u*, *v*, *w*) Virtual Temperature (*T*_*V*_)	0 to 20 ms^−1^ −35 to +55 °C	0.016 ms^−1^ 0.05 °C	50 Hz
	Inertial measurement unit	iµVRU + iTILT	iMAR	Attitude and motion	0 to 100 ms^−1^ 250° s^−1^	0.1°	50 Hz
Broadband radiation package (BP)	Pyrgeometers (2x)	CGR4	Kipp&Zonen	Upward, downward terrestrial irradiance (TIR)	−250 to +250 Wm^−2^ *λ* = 4.5 to 42 *μ*m	< ±7 Wm^−2^	12 Hz
	Pyranometers (2x)	CMP3	Kipp&Zonen	Upward, downward solar irradiance (*F*)	0 to 2000 Wm^−2^ *λ* = 0.3 to 2.8 *μ*m	<2 %	12 Hz
Cubic Aerosol Measurement Platform (CAMP)	Condensation Particle Counter (2x)	modified model 3007	TSI	Aerosol particle number concentration (*N*_8_, *N*_12_)	0 to 10^5^ cm^−3^ *D*_*p*_ = 0.008 to 2 *μ*m *D*_*p*_ = 0.012 to 2 *μ*m	10 % at averaging time > 10 s	1 Hz
	Optical particle size spectrometer	POPS	Handix	Aerosol particle number concentration (*N*_150_) and size distribution	0 to 1200 cm^−3^ *D*_*p*_ = 0.15 to 2.9 *μ*m	10 % at averaging time >10 s	1 Hz
	Absorption photometer	STAP 9406	Brechtel	Aerosol particle light absorption coefficient (*σ*_*abs*_)	> 0.2 Mm^−1^ *λ* = 450, 525, 624 nm	0.2 Mm^−1^ at averaging time > 60 s	1 Hz
Video Ice Particle Sampler (VIPS)	Video microscope	custom		Videos of cloud particles	*D*_*p*_ > 10 µm		12 Hz

The presented data set consists of six subsets, two for the BP and one for the other four instrument packages. The measured parameters feature profiles with instrument-dependent vertical resolutions of 0.01 m to 1 m, thus enabling a detailed assessment of the cloudy and cloud-free Arctic ABL above the sea ice in summer. From the data, it is possible to derive the general ABL structure, coupling state, extent of mixed layers, heating rates, presence of cloud droplets, and the vertical distribution of aerosol particle microphysical properties. The data set can be used as a framework to initialize numerical models and evaluate atmospheric processes, such as radiative effects of clouds, stratification, turbulence in the ABL, surface heat exchange, and in-cloud turbulent mixing.

## Methods

### BELUGA tethered balloon operations

**Fig. 2:**
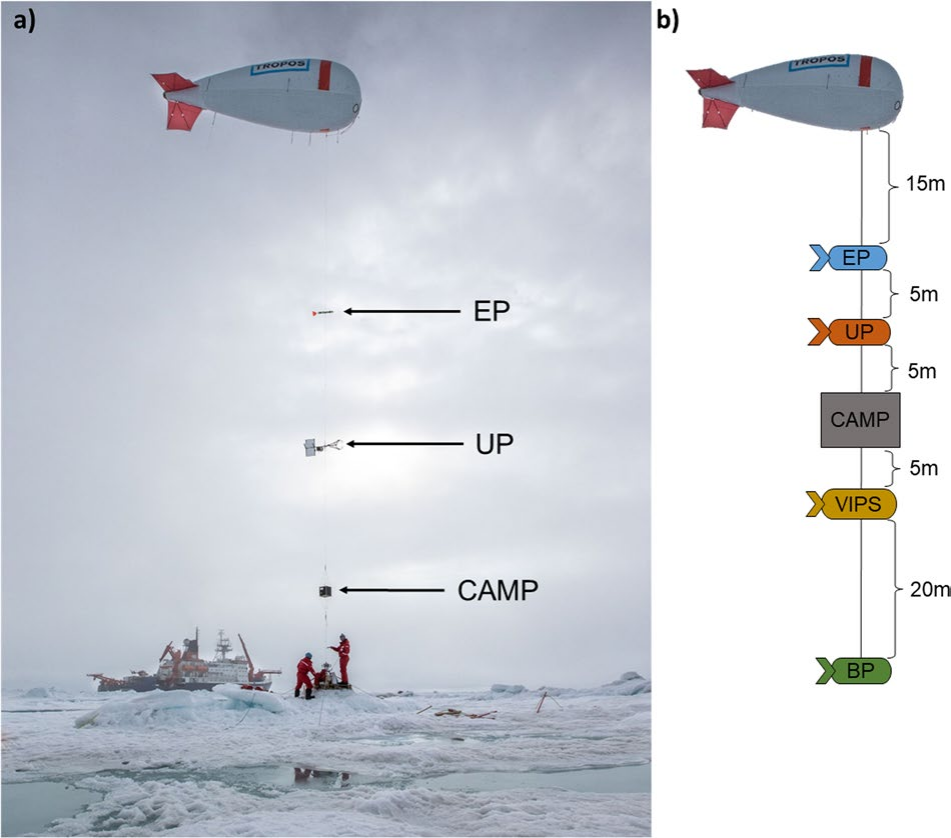
(**a**) Instruments attached to the tether of the balloon: extended meteorology package (EP), ultrasonic anemometer package (UP), and cubic aerosol measurement platform (CAMP). Photo credit: Lianna Nixon/CIRES at University of Colorado, Boulder. (**b**) Sketch of the typical instrument arrangement and dimensions; not all instruments were operated simultaneously.

The tethered balloon system BELUGA was previously deployed from an ice camp in the Arctic Ocean on the Physical feedbacks of Arctic PBL, Sea ice, Cloud, and AerosoL (PASCAL) campaign^14^. For MOSAiC, the BELUGA system was extended with a new balloon of the same size as the old version (filling volume 90 m^3^) but with the payload capacity increased from 15 kg to 20 kg due to new production materials. Because of technical issues, the new balloon was only used from 29 June to 15 July, and the old balloon was used from 19 to 27 July. The installations required for the balloon operations were located on the ice floe in “Balloon Town”, 260 m away from the starboard side of RV *Polarstern*^13^. A customized electrical winch system with a 3 mm thick and 2 km long tether with 9 kN strength (Dyneema) was used to operate the balloon up to a maximum altitude of 1.5 km. A turnable deflection pulley placed about 20 m away from the winch guided the tether. The balloon was connected to the tether by a 65 m custom-made sensor rope with integrated slings for instrument attachment. Varying combinations of the five instrument packages were operated with the specific positions on the tether and distances from each other, as shown in Fig. 2.

### Measurement strategy

The BELUGA operations were restricted to conditions with a maximum surface wind speed of less than 7.5 m s^−1^. The maximum payload capacity could be used up to 5 m s^−1^ with reduced payload and lower maximum flight altitudes for higher wind speed. Further limitations to available flight time were given by the general working hours on the ice floe, required balloon maintenance, and air space restrictions because of helicopter operations. The daily measurement strategy, including payload and flight altitude decisions, depended on the weather conditions and available time. If possible, at least one flight up to 1 km height for each instrument package was scheduled for each day of BELUGA operations, with the EP being obligatory on every flight. Combinations of more than two payloads in addition to the EP were only possible with the new balloon. Table 2 overviews all measurement flights and operated instruments.

**Table 2:**
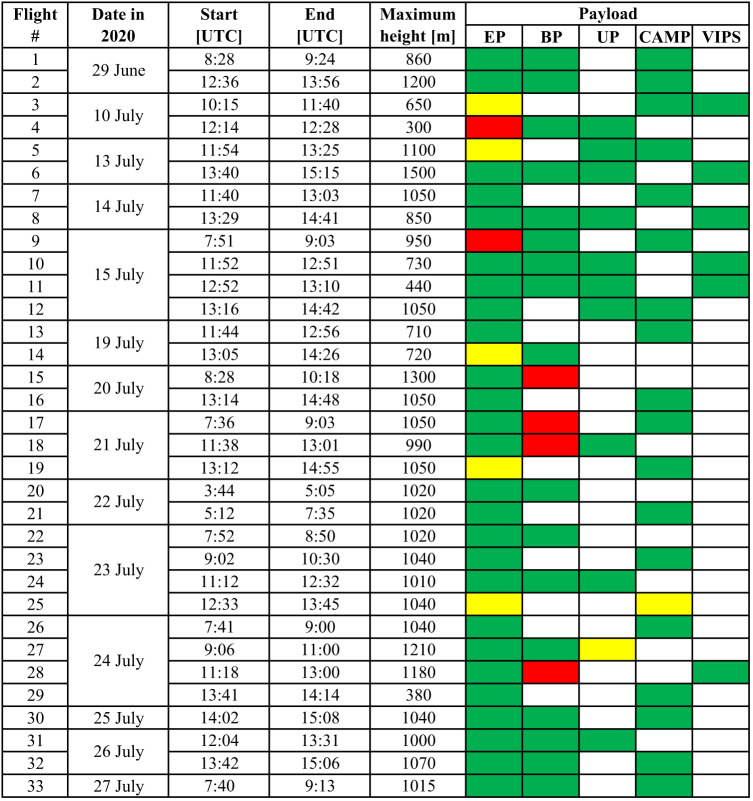
Overview of the BELUGA flights during MOSAiC with the operated payloads per flight in color coding . Operated instrument packages are color-coded in green, in yellow in case of a partial instrument failure, and in red in case of no data record.

### Flight strategy

**Fig. 3:**
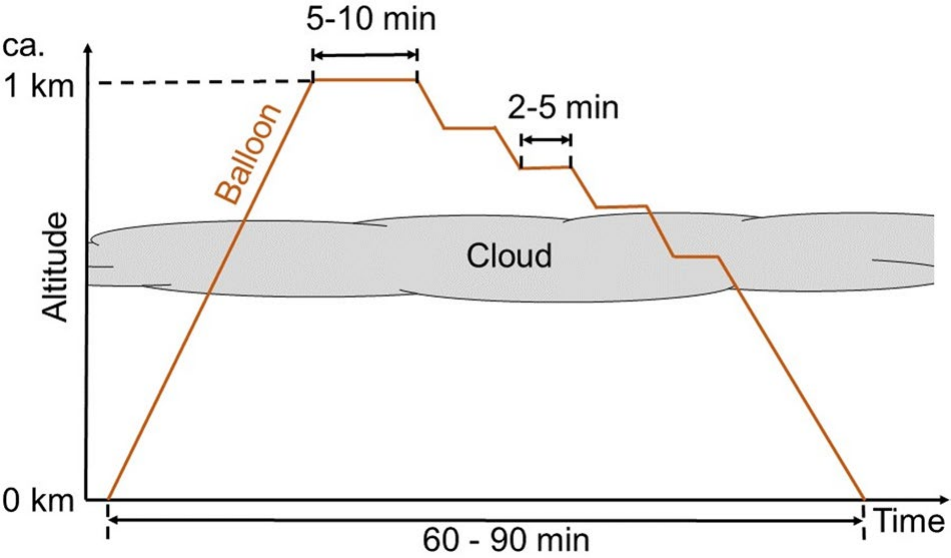
Typical balloon flight pattern illustrated by the altitude time series. A discontinuous descent follows a continuous ascent profile with constant altitude segments.

The balloon was operated in profiling mode with typical ascent and descent rates of 0.3 m s^−1^ to 1 m s^−1^ to achieve a high vertical resolution for measurements at 1 Hz. The atmospheric structure was identified during the continuous ascent by radio transmission of sensor data from the EP to a ground station. This information was used to adapt the downward-profile sampling strategy to the operated instruments, such as extended sampling time at the cloud top for turbulence and radiation measurements. An additional data down-link of particle number concentration from CAMP enabled extended sampling time in pronounced aerosol particle layers. Figure 3 displays an example of a balloon flight pattern as the altitude time series over a 60 min to 90 min time span, including 5 min to 10 min sampling periods at the maximum altitude and several stops of 2 min to 5 min during the descent.

### Extended meteorological package

**Fig. 4:**
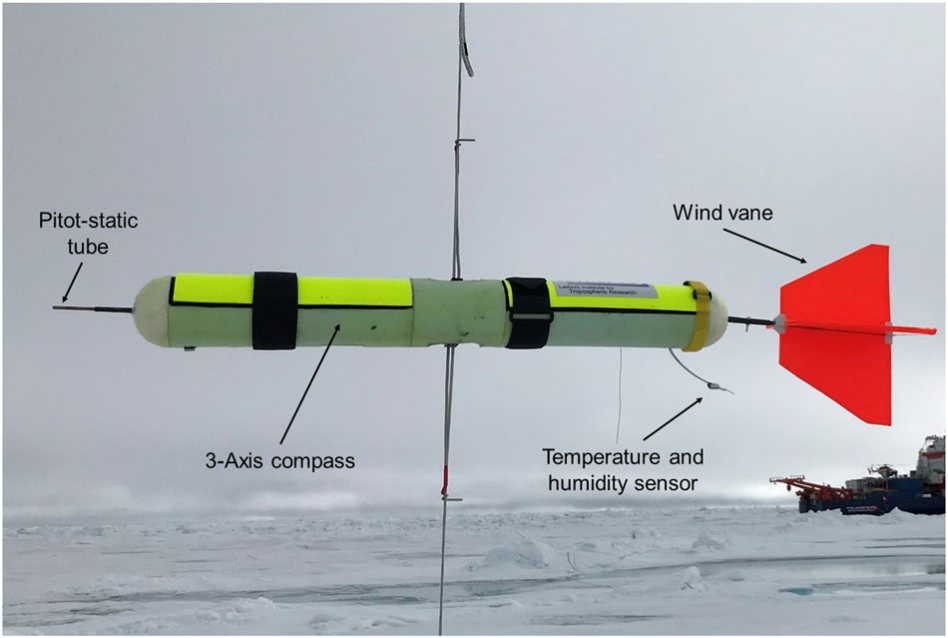
Extended meteorology package (EP).

A custom-built extended meteorological package (EP) of about 1 kg weight was developed and deployed during the BELUGA operations to measure basic meteorological parameters. The probe’s electronics are installed inside a custom-built housing with a wind vane on the rear end to ensure that the instrument points into the wind direction (Fig. 4). The thermodynamic properties temperature, relative humidity, and static air pressure, along with the position from the global navigation satellite system (GNSS), are measured at 1 Hz frequency with a standard radiosonde (DFM-17, GRAW). The horizontal wind speed is derived from the dynamic pressure measured with a Pitot-static tube at the front end of the probe. The orientation of the upwind pointing probe is recorded by an internal digital compass interpreted as wind direction. Both signals are processed by a microprocessor that generates wind speed and direction as serial data output that is integrated into the radiosonde data string. The entire data string is transmitted to a receiving station for monitoring.

### Ultrasonic anemometer package

**Fig. 5:**
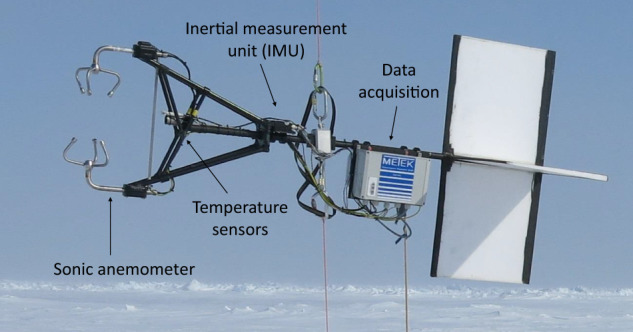
Ultrasonic anemometer package (UP).

The ultrasonic anemometer package (UP) measured the three-dimensional, Earth-referenced wind vector and the sonic (virtual) temperature with a sampling frequency of 50 Hz using a sonic anemometer (uSonic-3 Class A, Metek) with an external PT100 temperature sensor. The resolutions are 0.016 m s^−1^ and 0.05 °C, respectively. A flexible mount compensates for the tilt of the tether while attitude and motion are recorded by an inertial measurement unit (i*μ*VRU+iTILT, iMAR) for post-flight correction of the wind vector. A wind vane points the instrument upwind (Fig. 5). Further details on this instrument are provided by Egerer *et al*.^14^.

### Broadband radiation package

**Fig. 6:**
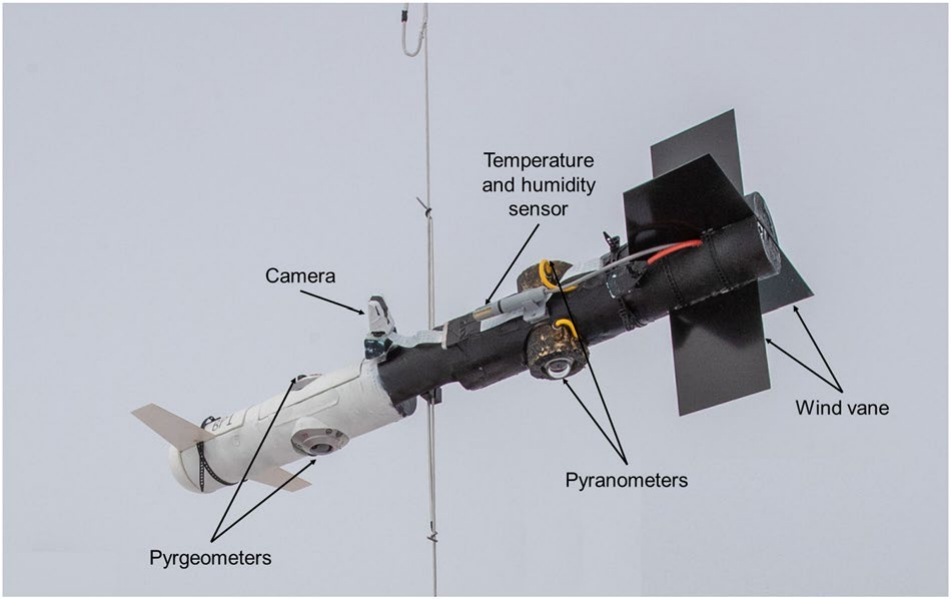
Broadband radiation package (BP).

The broadband radiation package (BP) is a custom-built probe of about 3 kg that measured upward and downward terrestrial and solar irradiances (Fig. 6). A pair of pyrgeometers (CGR4, Kipp & Zonen) sensitive to the spectral range from 4.5 *μ*m to 42 *μ*m measured the terrestrial irradiances, while a pair of pyranometers (CMP3, Kipp & Zonen) sensitive from 0.3 *μ*m to 2.8 *μ*m covered the solar spectrum. The radiometers were installed on upward and downward-facing cylinders connected by a main insulating body to reduce sensor icing. A flexible mount in the center of the main body served as the attachment on the tether. It enabled horizontal leveling while wind vanes on the front and rear end reduced pitching. An onboard IMU recorded the sensor’s attitude for corrections during post-processing. An external camera was installed to take pictures of the upper pyrgeometers at 0.1 Hz to enable the identification of liquid water or icing on the sensor domes. The BP was improved from the previous version^14^ by re-designing the probe’s main body, while the radiometers and data acquisition remained the same. The instrument was installed on the tether about 50 m below the balloon and at least 20 m below other payloads to avoid artificial shadowing effects on the pyranometers and limit the impact of the balloon temperature on measured terrestrial irradiances. The four radiometers on BP were cleaned regularly before each flight.

### Cubic aerosol measurement platform

**Fig. 7:**
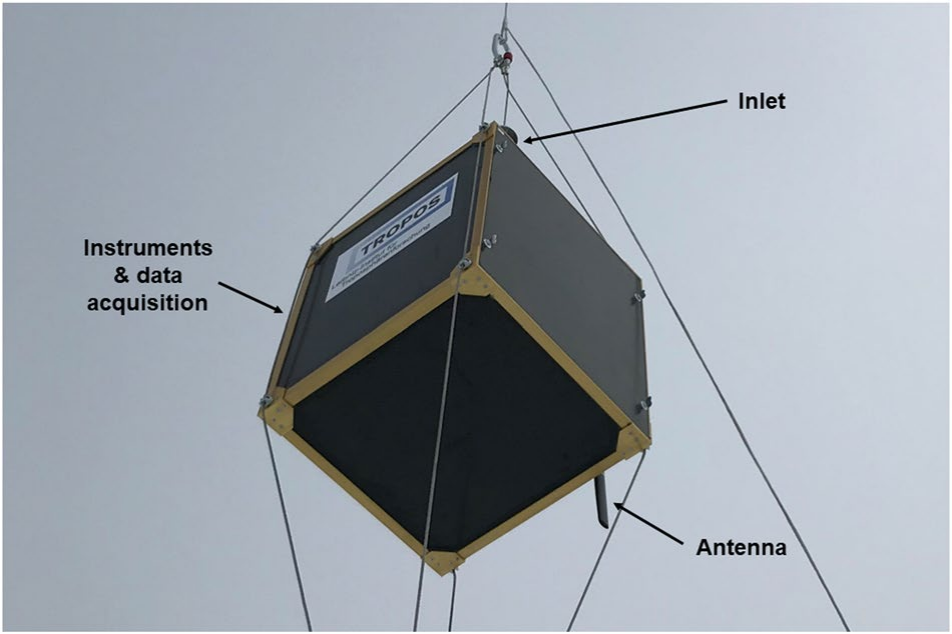
Cubic Aerosol Measurement Platform (CAMP).

The Cubic Aerosol Measurement Platform (CAMP) contained four aerosol instruments in an insulated housing with a temperature-controlled heating system (Fig. 7). Two modified condensation particle counters (CPC model 3007, TSI) measured particle number concentration with lower detection limits of 8 nm and 12 nm. An optical particle size spectrometer (POPS, Handix) recorded the optical particle number size distribution (PNSD) and particle number concentration in the size range from 0.15 *μ*m to 2.9 *μ*m in 14 size bins. The particle light absorption coefficient at 450 nm, 525 nm, and 624 nm wavelengths was measured by an absorption photometer (STAP model 9406, Brechtel).

A funneled inlet on top of CAMP sampled interstitial aerosol particles. A silica dryer installed upstream of the aerosol instruments ensured a sample air relative humidity below 40 %. A customized flow system with critical orifices and a vacuum scroll pump provided constant flow rates for the POPS and the two CPCs, thus achieving measurement uncertainties of ±10 % at averaging times above 10 s. The STAP’s lower detection limit was defined by the lowest achievable instrument noise of ±0.2 Mm^−1^ at averaging times larger than 60 s. Further details on CAMP and instrument characteristics can be found in Pilz *et al*.^15^.

The particle number concentration of the CPCs and the barometric pressure were radio-transmitted in real time to a receiving station during balloon flights. Before each balloon flight, the instruments were initialized and warmed up for at least 30 min with a high-efficiency particle filter installed at the inlet. The STAP was equipped with clean filters (371 M, Azumi) for each day of measurements.

### Video ice particle sampler

**Fig. 8:**
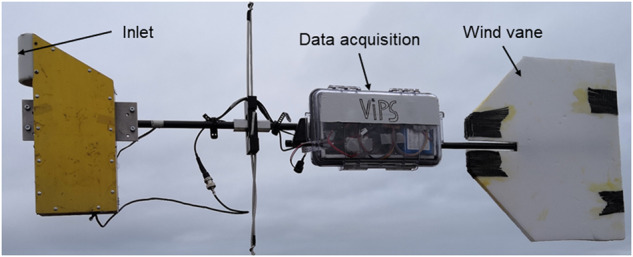
Video Ice Particle Sampler (VIPS) modified for balloon-borne measurements.

The Video Ice Particle Sampler (VIPS) recorded videos of cloud water droplets and ice crystals that were deposited on an 8 mm wide film covered in silicone oil with a video microscope^16,20^. Particles are imaged with a lower detection limit of 10 *μ*m. Adaptions were made for balloon-borne measurements from the previously used VIPS model on other airborne platforms^21,22^. The VIPS was installed on a carbon fiber boom with a self-balancing mechanism and a wind vane. A GPS sensor and a camera for imaging the sky in front of the instrument at 0.1 Hz were added (Fig. 8). The published VIPS data were flagged for the presence of cloud particles and their phase and included the observation height.

## Data Records

### Standard meteorological data

The processed meteorological data from the extended meteorology package (EP) published on PANGAEA^23^ (10.1594/PANGAEA.952341) contains 31 files in ASCII format. Each file contains the measurements of one flight, including an ascent and descent profile. The file names contain “BELUGA-Meteo_EP_“ followed by the start date and time (UTC) of the recording in “yyyy-mm-dd_hh-mm-ss” format. The files contain the following parameters: GNSS time, barometric pressure, temperature, relative humidity, horizontal wind speed, wind direction, and barometric altitude. The wind speed data is partially missing, particularly in clouds, due to the clogging of the Pitot-static tube by cloud particles. Due to an instrument malfunction, the EP data is unavailable on flights 4 and 9 on 10 and 15 July, respectively.

### High-resolution wind data

The high-resolution wind data from the ultrasonic anemometer package (UP) published on PANGAEA^24^ (10.1594/PANGAEA.931404) contains 8 files in ASCII format. The processed wind data are motion-corrected and provided in an Earth-referenced coordinate system. Each file contains the measurements of one day, thus multiple consecutive flights on 13 and 15 July. The file names contain “Balloon_Sonic_“ followed by the date, start, and end time (UTC) of measurement, in “yyyy-mm-dd_from_hh-mm_to_hh-mm” format. The files include the following parameters: GPS time, Longitude, Latitude, barometric altitude, GPS altitude, wind vector component in a west-east direction, wind vector component in south-north direction, vertical wind vector component (upward-directed), wind direction related to True North, barometric pressure, PT100 temperature (slow response), thermocouple temperature (fast response), and sonic (virtual) temperature. The ascent data are missing for flight 27 on 24 July, and the thermocouple data are unavailable for the entire day.

### Radiation data

The solar and terrestrial irradiances measured by the broadband radiation package (BP) are published in two separate data sets on PANGAEA due to their different levels of post-processing. Both data sets consist of 18 ASCII files, with each file containing data from one balloon flight, including an ascent and descent profile. In both data sets, flights 15, 17, 18, 28 on 20, 21, and 24 July are missing due to an instrument failure, and flight 33 was split into 2 files.

The processed terrestrial radiation data set^25^ (10.1594/PANGAEA.944200) was corrected for the sensor inertia removing the time lags and partly reconstructing fluctuations. The file naming consists of the flight number according to Table 2 as “Flight_#_” followed by “terrestrial_radiation.tab”. The files include the following parameters at 12 Hz resolution: UTC time, reconstructed downward and upward terrestrial irradiances, barometric altitude, pressure, temperature, relative humidity, and radiometer icing flag. Net irradiances can be calculated using upward and downward components, and heating rates can be derived^14^.

The raw solar radiation data set^26^ (10.1594/PANGAEA.944232) was not corrected for the sensor attitude because the level of complexity applied with the correction method depends on the intended data analysis (e.g., observations in cloudy or cloud-free conditions) and is therefore left to the data user. The file naming contains the flight number according to Table 2 as “Flight_#_“ followed by “solar_radiation.tab”. The files contain the following parameters at 12 Hz resolution: UTC time, uncorrected downward and upward solar irradiances, roll angle, pitch angle, yaw angle, barometric altitude, pressure, and radiometer icing flag.

### Aerosol data

The aerosol data measured by the Cubic Aerosol Measurement Platform (CAMP) published on PANGAEA^27^ (10.1594/PANGAEA.943907) contains 18 ASCII files. The single files contain the processed data of one flight, including an ascent and descent profile. The file names contain the first timestamp (UTC) at the beginning of measurements in “yyyy-mm-dd_hh-mm-ss” format followed by “_aerosol-particle-microphysics.tab”. Each file includes the following parameters at 1 Hz resolution: GNSS time, barometric altitude, barometric pressure, sample air temperature and relative humidity at the inlet (“out”) and downstream of the dryer (“in”), aerosol particle number concentration of the CPCs, integrated particle number concentration of the POPS, particle number size distribution of the POPS in 14 size bins as number concentration normalized by logarithmic bin width at the geometric mean particle diameter of each bin, and particle light absorption coefficients of the STAP. Local pollution effects on the descent profile of flight 30 on 25 July were filtered out. The POPS data is missing on flight 25 on 23 July due to an instrument malfunction.

Height-dependent number concentrations for different particle size ranges can be derived from the aerosol data. Particles in nucleation mode between 8 and 12 nm and Aitken mode between 12 and 150 can be derived from the difference in number concentrations of the two CPCs, and one CPC and the POPS, respectively. Particle surface, volume, and mass concentration in the accumulation and coarse mode can be derived from the POPS particle number size distribution, including conversion from optical to mobility diameter, as demonstrated in Pilz *et al*.^15^. An equivalent black carbon mass concentration can be derived from the absorption measurements by the STAP using a wavelength-dependent mass absorption cross-section.

### Cloud particle data

The published data set from the Video Ice Particle Sampler (VIPS)^28^ (10.1594/PANGAEA.944068) contains the following parameters at 12 Hz resolution: UTC time, height, and water detection flag. It was derived by flagging the occurrence of water drops as seen in the raw videos imaging the moving film.

## Technical Validation

### Meteorological data

The observations by the extended meteorology package (EP) near the ground (below 10 m height) were validated and corrected by reference measurements of a meteorological tower^29^ operated by the University of Colorado/NOAA flux team at Met City near Balloon Town for every balloon flight. Temperature offsets resulting from the probe’s handling were corrected. Occasionally recorded RH values above 100 % inside clouds probably resulted from sensor wetting and were corrected to 100 %. This assumption was supported by the cloud top derived from the terrestrial irradiance measurements on BELUGA and by comparing ascent with descent profiles. The wind speed was calibrated in laboratory experiments and validated with the Met City observations during data processing.

### High-resolution wind data

The ultrasonic anemometer package (UP) recorded two raw data files, one from the sonic anemometer itself and one from the IMU, both at 50 Hz. Custom processing software was used to convert the binary IMU data to an ASCII format before both data streams were merged and synchronized by the extracted timestamps from both files. The wind velocity vector is initially measured in a reference system fixed to the ultrasonic anemometer. By means of the attitude angles and velocity components estimated with the IMU, the wind vector is transformed into an Earth-fixed system (see Egerer *et al*.^14^ for more details). Possible offset angles between the ultrasonic anemometer and the IMU can be estimated by assuming a vanishing mean vertical wind averaged over a sufficient period (here 5 min are used). The instrument motion was eliminated almost completely in most parts of the wind data, except periods at low altitudes with strong yaw oscillations of the instrument (up to 70°, f = 0.2 Hz). In the final processing step, the wind coordinate system was changed from north-east-down to east-north-up to comply with PANGAEA conventions. The temperature measurements were corrected for their time response (PT100 ca. 10 s, thermocouple ca. 0.64 s) and calibrated as described in more detail by Egerer *et al*.^14^.

**Fig. 9:**
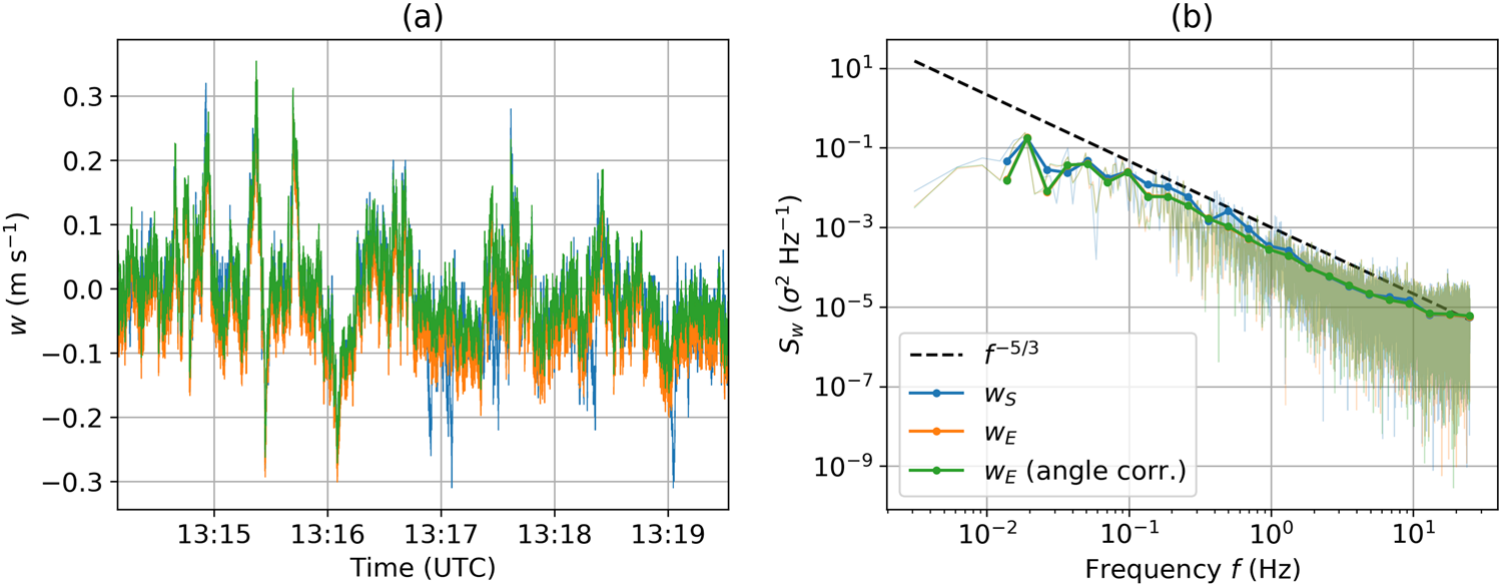
(**a**) Time series of the vertical wind component measured by the UP during calibration at a constant altitude close to the surface and (**b**) power spectral density for this time period on 15 July 2020. Both subplots show the raw wind speed as measured by the sonic anemometer w_*s*_ (blue), and motion-corrected vertical wind velocity w_*E*_ without (orange) and with (green) correction of the installation angle offsets between sonic and IMU. The green lines represent the data of the published dataset.

The quality of the wind vector transformation and correction was tested with a 5 min time series of the vertical wind component *w* and its power spectral density (PSD) recorded at a constant altitude close to the surface (Fig. 9). In the displayed time series, the instrument motion shows in the uncorrected turbulent wind velocity *w*_*s*_, resulting in multiple PSD peaks between 0.2 Hz and 2 Hz. These peaks, hence the instrument motion, are almost entirely eliminated in the corrected vertical wind velocity w_*E*_ spectrum. The offset angle correction does not notably impact the spectrum but averages the mean wind velocity over the calibration period to zero. After corrections, the spectrum exhibits a −5/3 slope (indicating inertial sub-range scaling) in a frequency range between 5·10^−2^ Hz to around 10 Hz. The spectral noise floor is below 10^−5^ *σ*^2^ Hz^−1^, indicating a standard deviation due to uncorrelated noise of 0.016 m s^−1^.

### Radiation data

The radiometers on the broadband radiation package (BP) were operated without ventilation and radiation shielding systems because of payload limitations, assuming that the in-flight wind on the sensors led to a quick adjustment to changes in the outside temperature. Thus, the effect of varying radiometer body temperatures is covered by the temperature compensation derived from calibrations when remaining in the operational range. The radiometers were used at temperatures between −15 °C and 15 °C, well above the operational minimum (−40 °C) indicated by the manufacturer. In these conditions, the non-linearity of the sensor calibration was below 1 %.

A cross-calibration of the radiometers was performed at the regional radiation center at Lindenberg Observatory, Germany. The pyrgeometers (measurement range 4.5 *μ*m to 42 *μ*m) were calibrated for the full range of terrestrial irradiance from 3 *μ*m to 100 *μ*m, and the pyranometers (measurement range 0.3 *μ*m to 2.8 µm) to the entire solar range from 0.29 *μ*m to 3 *μ*m.

The installation of the pyrgeometers on the opposing sides of a common metal body has a negligible impact on sensor characteristics^30^. Besides the reduced weight, this system has the advantage of balancing the temperature of the two pyrgeometers. The equal temperature biases remove the temperature offset when calculating the net terrestrial irradiance. Offsets between the upper and the lower sensor were estimated by measurements at the ground, comparing measurements when turning the two radiometers over with respect to the original orientation. The analysis found that the offsets were below 1 Wm^−2^.

Operating radiometers inside clouds potentially leads to the presence of ice or liquid water on the sensor domes. The occurrence of icing on the upper pyrgeometers was identified by an external camera on BP taking pictures of them at 0.1 Hz and flagged in the data during post-processing. Partial icing was observed on 8 of 18 flights, usually during ascents at the cloud-top region and on descents inside and below the cloud.

**Fig. 10:**
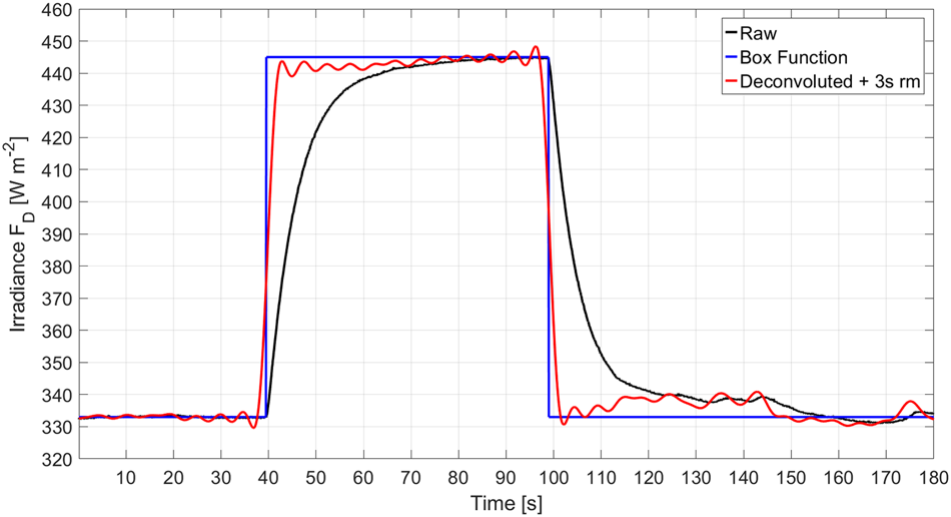
Reconstruction of measured terrestrial irradiances. The raw irradiance (black line) has a slow response to a box function (blue line). Observations are corrected for the response time of the pyrgeometer using a deconvolution method and then smoothed with a 3 s running mean (red line). The remaining oscillations in the corrected signal result from the Gibbs phenomenon associated with the Fourier transform.

Laboratory tests determined the inversely exponential response time of the pyranometers and pyrgeometers on BP to be less than stated by the manufacturer to 2 s and 6 s, respectively. Such response times lead to an underestimation of natural fluctuations in the irradiances, for instance, at the cloud top. A correction to the terrestrial irradiances was performed with a deconvolution algorithm^31^ using a Fourier transform with a cut-off frequency of 0.2 Hz and a moving mean of 1 s. Deconvoluted data were further averaged with a moving mean of 3 s to reduce the noise due to the Gibbs phenomenon associated with the Fourier transform in the deconvolution algorithm as shown in Fig. 10. The remaining oscillations are in the order of ± 3 Wm^−2^ in correspondence with the edges of the box function, and below ± 1 Wm^−2^ elsewhere. The total uncertainty of reconstructed terrestrial irradiances is estimated to be below ± 7 Wm^−2^. Measurements at the surface without sensor movements were found to be in close agreement (± 3 Wm^−2^) with surface observations made by the Atmospheric Surface Flux Station^32^ and a radiation station operated by the U.S. Department of Energy Atmospheric Radiation Measurement (ARM) Program^33^ at Met City near Balloon Town^17^.

For the solar irradiances, the measurement uncertainty was derived from observed high-frequency noise that was induced by payload movements. A 2 % uncertainty was estimated for measurements in overcast conditions. Direct comparisons with surface observations were impossible due to the effects of inhomogeneous surface albedo at the different sites and the high temporal and spatial variability of cloud cover.

### Aerosol data

Laboratory calibrations of the aerosol instruments on CAMP were performed before and after the MOSAiC measurements. The detection limits and counting efficiencies of the CPCs were determined with size-selected silver particles against a reference Electrometer. The POPS’s sizing accuracy and counting efficiency were calibrated with size-selected polystyrene latex spheres (PSL) against a reference CPC. The detection limit of the STAP was determined with long-term measurements of particle-free air. A 25 % enhancement of absorption values by the STAP with the used Azumi filters was found in laboratory comparisons. More details on instrument characterization, calibration, and sampling system losses can be found in Pilz *et al*.^15^.

During MOSAiC, CAMP recorded three data files, one for the STAP, one for the POPS, and one for the two CPCs. The CPC data file also contained barometric pressure, sample air temperature, and relative humidity downstream of the inlet and dryer, the inlet pressure of the CPCs, and the GNSS time and position. The three data files were synchronized by the individually recorded inlet pressures, referenced to the barometric pressure, and merged into one single file. Subsequently, the measurements were corrected for instrument-dependent counting efficiencies and particle losses of the sampling system. Finally, corrections to a standard temperature of 273.15 K and pressure of 1013.25 hPa, and checks for local pollution were applied to the data before publishing on PANGAEA.

**Fig. 11:**
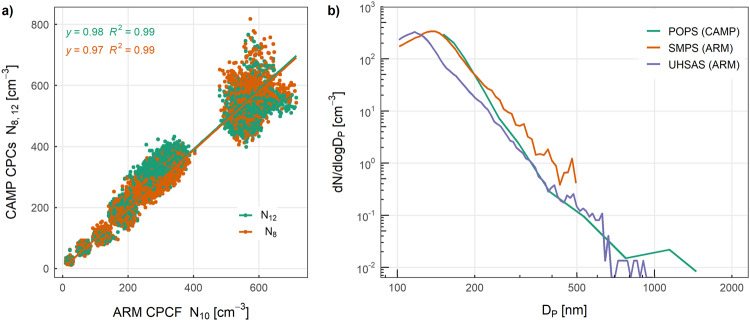
Comparison of (**a**) particle number concentrations measured by CAMP CPCs with ARM CPCF, and (**b**) two-hour mean PNSD by CAMP POPS, ARM SMPS, and ARM UHSAS on 14 July 2020. The SMPS measured the mobility PNSD between 10 and 500 nm at a 5 min scanning time. The UHSAS measured the PNSD between 60 and 1000 nm at an optical scattering diameter of 1054 nm every 10 s.

The processed CAMP data were evaluated against the aerosol observations made by the ARM Program onboard RV *Polarstern*. Time series of CAMP measurements at the surface at Balloon Town, either before or after a balloon flight, were compared with the ARM measurements, excluding terms of local pollution. The comparison periods distributed over 12 days during the BELUGA observations typically varied between 10 min and 30 min per day with a total length of 5 h. Particle number concentrations measured by the CAMP CPCs (*N*_8_ and *N*_12_) were on average 3 % and 2 % lower (R^2^ = 0.99) than the ARM CPCF^34^ with a detection limit of 10 nm (*N*_10_) (Fig. 11a)). A comparison of a two-hour mean PNSD measured by the POPS when CAMP was at the surface at Balloon Town with the ARM Ultra-High Sensitivity Aerosol Spectrometer (UHSAS^35^) and the ARM Scanning mobility particle size spectrometer (SMPS^36^) onboard RV *Polarstern* showed reasonable agreement on 14 July 2020 (Fig. 11b)).

## Data Availability

The custom software and codes used to process the published data set of the EP^37^, the UP^38^, the BP^39^, and CAMP^40^ are publicly available on zenodo.org.

## References

[1] Box JE (2019). Key indicators of Arctic climate change: 1971–2017. Environmental Research Letters.

[2] Rantanen M (2022). The Arctic has warmed nearly four times faster than the globe since 1979. Communications Earth & Environment.

[3] Serreze MC, Barry RG (2011). Processes and impacts of Arctic amplification: A research synthesis. Glob. Planet. Change.

[4] Pithan F, Mauritsen T (2014). Arctic amplification dominated by temperature feedbacks in contemporary climate models. Nature Geoscience.

[5] Wendisch M (2019). The Arctic cloud puzzle using ACLOUD/PASCAL multiplatform observations to unravel the role of clouds and aerosol particles in Arctic amplification. Bulletin of the American Meteorological Society.

[6] Shupe MD (2013). Cloud and boundary layer interactions over the Arctic sea ice in late summer. Atmos. Chem. and Phys..

[7] Brooks IM (2017). The turbulent structure of the Arctic summer boundary layer during the Arctic Summer Cloud-Ocean Study. Journal of Geophysical Research: Atmos..

[8] Turner DD, Shupe MD, Zwink AB (2018). Characteristic atmospheric radiative heating rate profiles in Arctic clouds as observed at Barrow, Alaska. Journal of Applied Meteorology and Climatology.

[9] Morrison H (2012). Resilience of persistent Arctic mixed-phase clouds. Nature Geoscience.

[10] Mauritsen T (2011). An Arctic CCN-limited cloud-aerosol regime. Atmos. Chem. and Phys..

[11] AMAP, 2015. AMAP Assessment 2015: Black carbon and ozone as Arctic climate forcers. Arctic Monitoring and Assessment Programme (AMAP), Oslo, Norway. vii + 116 pp. https://www.amap.no/documents/doc/amap-assessment-2015-black-carbon-and-ozone-as-arctic-climate-forcers/1299 (2015).

[12] Knust R (2017). Polar research and supply vessel Polarstern operated by the Alfred-Wegener-Institute. Journal of Large-Scale Research Facilities.

[13] Shupe MD (2022). Overview of the MOSAiC expedition: Atmosphere. Elementa: Science of the Anthropocene.

[14] Egerer U, Gottschalk M, Siebert H, Ehrlich A, Wendisch M (2019). The new BELUGA setup for collocated turbulence and radiation measurements using a tethered balloon: First applications in the cloudy Arctic boundary layer. Atmos. Meas. Tech..

[15] Pilz C (2022). CAMP: an instrumented platform for balloon-borne aerosol particle studies in the lower atmosphere. Atmos. Meas. Tech..

[16] Heymsfield AJ, McFarquhar GM (1996). High albedos of cirrus in the tropical Pacific warm pool: Microphysical interpretations from CEPEX and from Kwajalein, Marshall Islands. Journal of Atmos. Sciences.

[17] Lonardi M (2022). Tethered balloon-borne profile measurements of atmospheric properties in the cloudy atmospheric boundary layer over the Arctic sea ice during MOSAiC: Overview and first results. Elementa: Science of the Anthropocene.

[18] Angot H (2022). Year-round trace gas measurements in the central Arctic during the MOSAiC expedition. Scientific Data.

[19] de Boer G (2022). Observing the central Arctic atmosphere and surface with University of Colorado uncrewed aircraft systems. Scientific Data 2022 9:1.

[20] McFarquhar GM, Heymsfield AJ (1996). Microphysical characteristics of three anvils sampled during the central equatorial pacific experiment. Journal of Atmos. Sciences.

[21] Schmitt CG, Heymsfield AJ (2009). The size distribution and mass-weighted terminal velocity of low-latitude tropopause cirrus crystal populations. Journal of the Atmos. Sciences.

[22] Schmitt CG (2013). The microphysical properties of ice fog measured in urban environments of interior Alaska. Journal of Geophysical Research: Atmos..

[23] Pilz C, Siebert H, Lonardi M (2022). PANGAEA.

[24] Egerer U, Pilz C, Lonardi M, Siebert H, Wendisch M (2021). PANGAEA.

[25] Lonardi M, Pilz C, Siebert H, Ehrlich A, Wendisch M (2022). PANGAEA.

[26] Lonardi M, Pilz C, Siebert H, Ehrlich A, Wendisch M (2022). PANGAEA.

[27] Pilz C, Lonardi M, Siebert H, Wehner B (2022). PANGAEA.

[28] Lonardi M, Pilz C, Siebert H, Ehrlich A, Wendisch M (2022). PANGAEA.

[29] Cox C (2021). Arctic Data Center.

[30] Becker R, Maturilli M, Philipona R, Behrens K (2020). *In situ* sounding of radiative flux profiles through the Arctic lower troposphere. *Bulletin of Atmos*. Science and Technology.

[31] Ehrlich A, Wendisch M (2015). Reconstruction of high-resolution time series from slow-response broadband terrestrial irradiance measurements by deconvolution. Atmos. Meas. Tech..

[32] Cox C (2021). Arctic Data Center.

[33] Riihimaki, L. Radiation instruments on ice (ICERADRIIHIMAKI). Oct 2019 - Oct 2020, MOSAIC (Drifting Obs - Study of Arctic Climate); Mobile Facility (MOS). *Atmospheric Radiation Measurement (ARM) user facility Data Center*10.5439/1814821.

[34] Koontz A, Kuang C (2022). Atmospheric Radiation Measurement (ARM) user facility Data Center.

[35] Uin J, Senum G, Koontz A, Flynn C (2022). Atmospheric Radiation Measurement (ARM) user facility Data Center.

[36] Kuang C, Singh A, Howie J (2022). Atmospheric Radiation Measurement (ARM) user facility Data Center.

[37] Siebert H (2023). Zenodo.

[38] Egerer U, Siebert H (2023). Zenodo.

[39] Lonardi M (2023). Zenodo.

[40] Pilz C, Düsing S (2023). Zenodo.

[41] Nixdorf U (2021). Zenodo.

[42] Ludwig V, Spreen G, Pedersen LT (2020). Evaluation of a new merged sea-ice concentration dataset at 1 km resolution from thermal infrared and passive microwave satellite sata in the Arctic. Remote Sensing.

